# U. S. V. M. A.

**Published:** 1898-05

**Authors:** 


### SOCIETY PROCEEDINGS.

### U. S. V. M. A.

THE opening of the discussion on meat-inspection has been arranged as follows :

"Reasons for Meat-inspection," Dr. C. A. Cary.

"Methods of Educating the Public as to the Importance of Meat-inspection," Dr. W. Horace Hoskins.

"The Necessity of Consolidation of Municipal Slaughter-houses into Large Abattoirs under Municipal Control," Dr. Leonard Pearson.

"Slaughter-house Inspection," Dr. Thomas J. Turner.

"Retail Market Inspection," Dr. Charles W. Heitzman.

In connection with the discussion there will be displayed a large variety of pathological conditions and diseased tissues in demonstration of diseases found in slaughter-houses and market inspection.

This subject ought to prove of the greatest importance and interest to veterinarians in all our cities and towns, and aid in securing legislation needed for this much-to-be-desired purpose among our people.

The joint meetings of the Nebraska and Iowa associations in connection with the U. S. V. M. A. will be of a social character and add to the fraternal spirit of the occasion.

The Nebraska Association proposes to supply subjects for a number of operations during the sessions. These to be performed from 8 to 10 A.M., by leading surgeons from among the members. It is to be hoped that volunteers will be prompt in responding to this call, and that such operations as arytenectomy, oöphorectomy in the mare, castration of cryptorchids, median neurectomy, and the several operations for stringhalt may be performed. A suitable place has been arranged for close to the assembly rooms of the convention. Papers on surgical subjects are desired, and if these can be demonstrated clinically it is thought that much benefit may accrue to those in attendance.

Papers are already announced from Drs. Law, Cary, T. S. Butler, R. R. Bell, and A. J. Anderson.

